# Value of radiomics-based two-dimensional ultrasound for diagnosing early diabetic nephropathy

**DOI:** 10.1038/s41598-023-47449-2

**Published:** 2023-11-22

**Authors:** Xuee Su, Shu Lin, Yinqiong Huang

**Affiliations:** 1 Centre of Neurological and Metabolic Research, The Second Affiliated Hospital of Fujian Medical University, Quanzhou, Fujian Province China; 2 Department of Anaesthesia, The Second Affiliated Hospital of Fujian Medical University, Quanzhou, Fujian Province China; 3 Diabetes and Metabolism Division, Garvan Institute of Medical Research, 384 Victoria Street, Darlinghurst, Sydney, NSW 2010 Australia; 4 Present Address: Department of Endocrinology, The Second Affiliated Hospital of Fujian Medical University, Quanzhou, Fujian Province China

**Keywords:** Computational biology and bioinformatics, Endocrinology

## Abstract

Despite efforts to diagnose diabetic nephropathy (DN) using biochemical data or ultrasound imaging separately, a significant gap exists regarding the development of integrated models combining both modalities for enhanced early DN diagnosis. Therefore, we aimed to assess the ability of machine learning models containing two-dimensional ultrasound imaging and biochemical data to diagnose early DN in patients with type 2 diabetes mellitus (T2DM). This retrospective study included 219 patients, divided into a training or test group at an 8:2 ratio. Features were selected using minimum redundancy maximum relevance and random forest-recursive feature elimination. The predictive performance of the models was evaluated using the area under the receiver operating characteristic curve (AUC) for sensitivity, specificity, Matthews Correlation Coefficient, F1 score, and accuracy. K-nearest neighbor, support vector machine, and logistic regression models could diagnose early DN, with AUC values of 0.94, 0.85, and 0.85 in the training cohort and 0.91, 0.84, and 0.84 in the test cohort, respectively. Early DN diagnosing using two-dimensional ultrasound-based radiomics models can potentially revolutionize T2DM patient care by enabling proactive interventions, ultimately improving patient outcomes. Our integrated approach showcases the power of artificial intelligence in medical imaging, enhancing early disease detection strategies with far-reaching applications across medical disciplines.

## Introduction

The economic and health burden of diabetes mellitus (DM) is increasing globally owing to the aging population and lifestyle changes. DM affects approximately 425 million people globally and is expected to affect 693 million adults by 2045^1^. Type 2 DM (T2DM) accounts for approximately 90–95% of DM cases^2^. People with T2DM are at an increased risk of developing diabetic nephropathy (DN) and diabetic retinopathy. The prevalence of DN in patients with T2DM is 30–40%, and DN accounts for 30–47% of new end-stage renal disease (ESRD) cases, increasing mortality from T2DM^3^. Mortality from DN increases with disease progression^4^. Therefore, the early diagnosis of DN is essential for improving patient survival and quality of life.

At present, proteinuria and reduced glomerular filtration rate are used for the early diagnosis of DN^5^. However, using these markers to diagnose DN is controversial^6–9^. With the development of ultrasound techniques, ultrasound has been the most important technique used to detect DN in clinical practice, especially two-dimensional (2D) ultrasound, because of its simplicity, speed, and non-invasive nature^10^. Characteristic changes, such as kidney length, cortical medulla differentiation, and blood flow, allow interpretation of kidney lesions and avoid the performance of invasive procedures, such as renal biopsy^11^. However, due to the quality of ultrasound images, 2D ultrasound use can be difficult in the diagnosis of early DN.

Radiomics is a process designed to extract high-throughput information from medical images to support diagnostic decision making, whereby a large number of collected features are used in machine learning to distinguish tissue types^12,13^. Radiomics has been applied to different imaging modalities for the identification and differentiation of renal diseases, including the study of radiomics features of chronic kidney disease^14^. Diagnosis of chronic kidney disease based on ultrasound^15^, detect fibrosis in chronic kidney disease^16^, assess renal fibrosis in patients with chronic kidney disease^17^, and evaluate the function of transplanted kidneys^18,19^. However, the risk reduction of ESRD awaits the realization of an early diagnosis. A previous study showed that early diagnosis of DN will result in an 80% lower risk in ESRD progression, with important implications for patient outcomes and reducing global health burden^20^.

Given the above points, we intended to explore the radiomics data of 2D ultrasound images, combined with blood biochemical indexes, to construct a diagnostic model of early DN.

## Materials and methods

### Patients and ethics approval

This study was conducted in accordance with the Declaration of Helsinki and approved by the Medical Ethics Committee of the Second Affiliated Hospital of Fujian Medical University (Approval No. 2023252). Due to the retrospective nature of the study, the need of informed consent was waived by the Medical Ethics Committee of the Second Affiliated Hospital of Fujian Medical University.

In this retrospective study, we enrolled 219 patients admitted to the Department of Endocrinology of the Second Affiliated Hospital of Fujian Medical University from July 1, 2017, to May 1, 2023. All patients underwent 2D ultrasound examination. The routine ultrasound examination procedure was performed as follows: patients were placed in the supine position, with clear kidney exposure, and good-quality images were obtained, including complete renal length diameter, renal transverse diameter, and renal parenchymal thickness.

### Diagnosis

In our cohort, 114 patients were diagnosed with T2DM without DN, and 105 patients were diagnosed with early DN by renal biopsy. The early-stage DN was defined as a DN stage lower than or equal to stage III, categories G1A1–G3A3.

### Inclusion and exclusion criteria

The inclusion criteria were (1) patients with uncomplicated T2DM; (2) patients with DN (categories G1A1–G3A3) diagnosed by a nephrologist or patients with DN (stages I–III) diagnosed by renal biopsy using the 2012 Kidney Disease: Improving Global Outcomes (KDIGO) clinical practice guideline for the evaluation and management of chronic kidney disease (CKD) based on the cause, GFR, and albuminuria system and the Renal Pathology Society’s consensus classification of DN (RPS)^21,22^; and (3) 2D ultrasound images and biochemical data were collected during the study period.

The exclusion criteria were (1) patients with CKD not caused by T2DM; (2) patients with kidney tumors, stones, and cysts; (3) patients with low-quality images; and (4) patients with missing data.

### Demographic and clinical information

**Figure 1:**
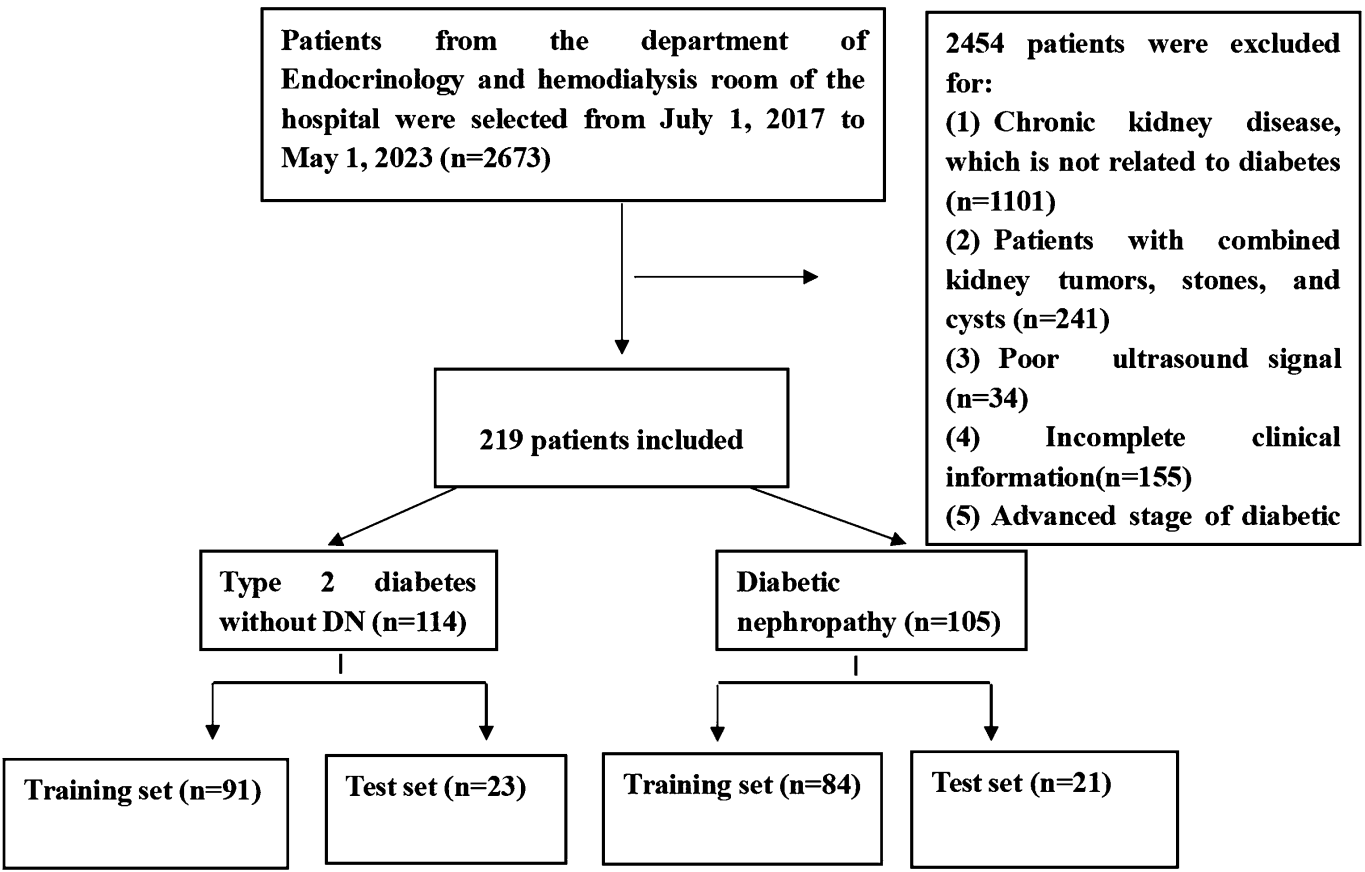
Flow diagram of the included participants.

The following demographic and clinical information were collected: age, sex, total cholesterol (TC), triglycerides (TG), high-density lipoprotein cholesterol (HDL-C), low-density lipoprotein cholesterol (LDL-C), apolipoprotein (apo) A (ApoA), ApoB, blood urea nitrogen (BUN), creatinine (CREA), BUN/CREA, uric acid (UA), and blood glucose (GLU) (Fig. 1 and Supplementary Table S1).

### Ultrasound features and diagnostic accuracy

Radiomics extracts many imaging features, including first-order features, shape, texture, gray level co-occurrence matrix, and gray level size zone matrix, which can be analyzed by machine learning algorithms^23^. Quantitative features obtained from ultrasound images include echogenicity, shapes, shear wave velocity, intrarenal artery peak systolic velocity, end-diastolic velocity, resistive index, and organ size^24,25^. We used random forest (RF) to determine the optimal combination of radiomics features to distinguish between healthy and diseased kidneys. Moreover, we performed five-fold cross-validation over the entire dataset to determine optimal parameters. The diagnostic accuracy of each model was evaluated using the area under the receiver operating characteristic curve (AUC) for sensitivity, specificity, Matthews Correlation Coefficient (MCC), F1 score, and accuracy.

### Kidney segmentation

**Figure 2:**
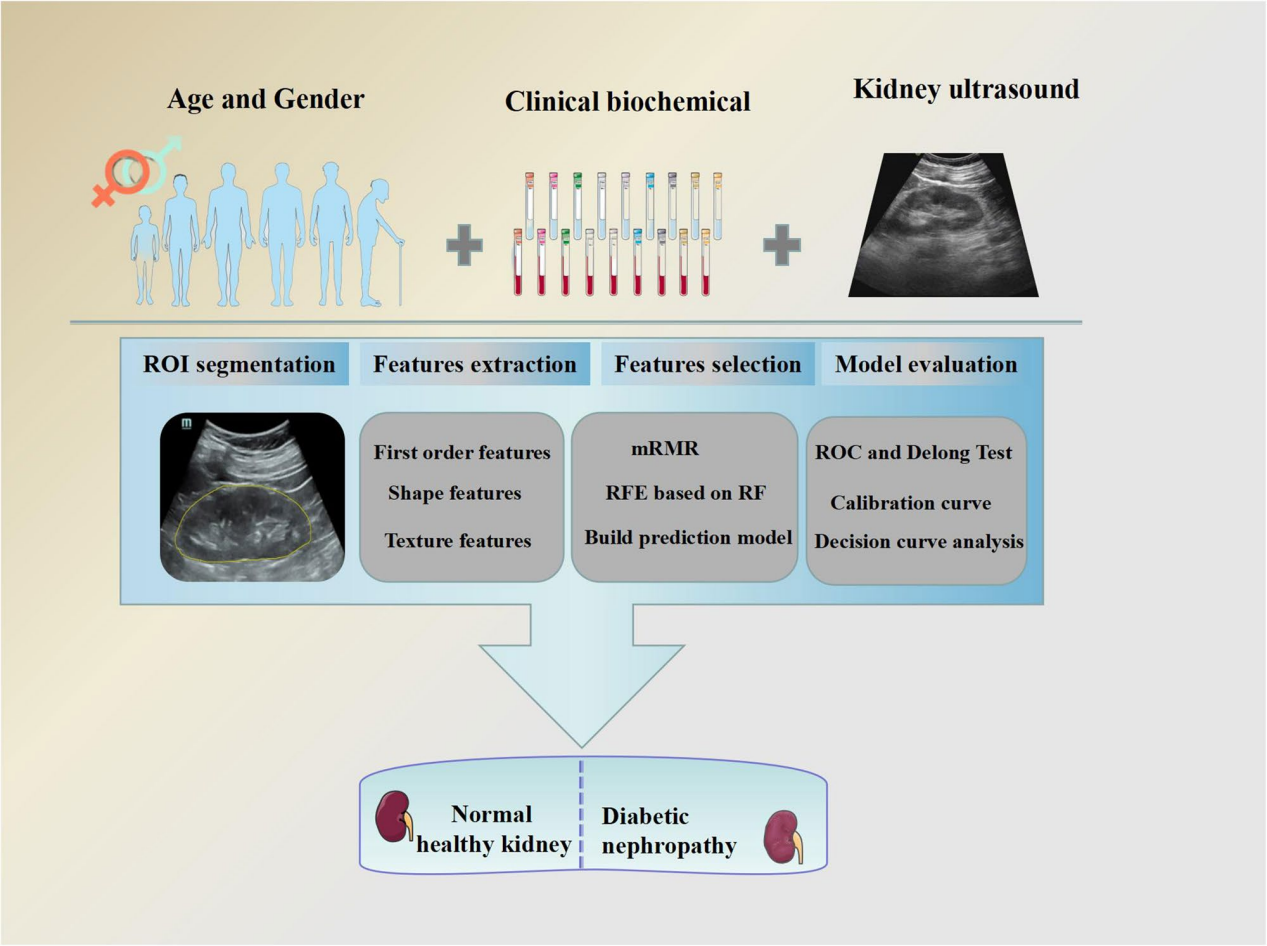
The general workflow of this study.

**Figure 3:**
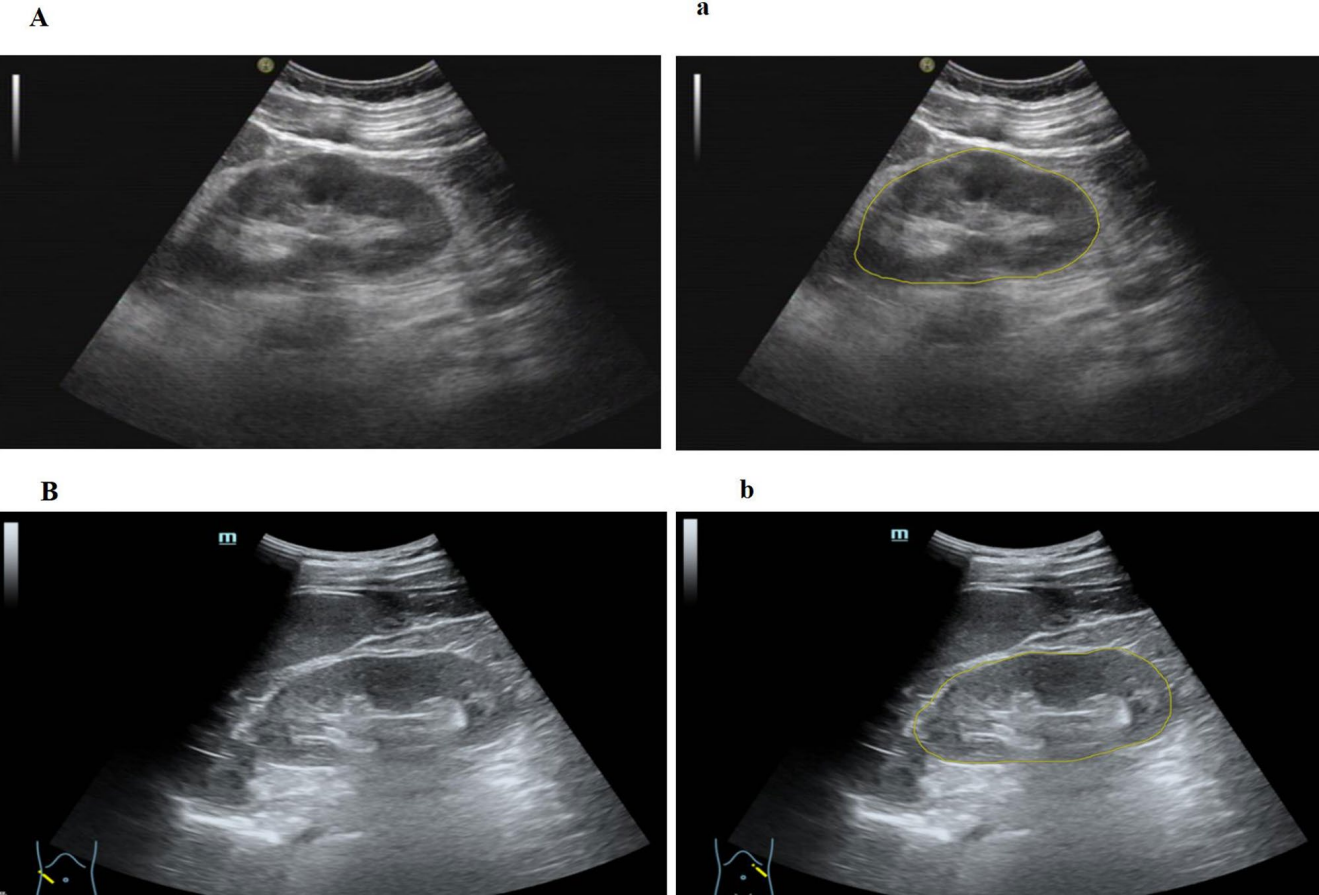
Representative plot of region of interest segmentation in patients with diabetic nephropathy (**A**) and diabetic patients without renal complications (**B**). (**a**) Raw grayscale image, (**b**) segmentation of the kidney region of interest.

Ultrasound images and clinical data were imported into the Darwin research platform using “Darwin research platform” software developed by Yi Zhun Medical (https://www.yizhun-ai.com). The flowchart of image processing is illustrated in Fig. 2. Regions of interest (ROIs) were selected by a physician with 5 years of experience in ultrasound. The largest coronal section of the kidney was collected, and the ROI was placed on the kidney (Fig. 3). Another sonographer with 10 years of experience in ultrasound examined the segmentation results. Disagreements were resolved by consensus.

### Radiomics feature extraction and selection

Feature extraction was performed using the radiomics software package (Python version 3.6.9). The software implements algorithms that calculate a wide range of features, including first-order statistics, shape-based features, texture-based features (e.g., GLCM, GLRLM, or wavelet-based features), and higher-order statistical features. After extracting a large number of radiomics features, a subset of relevant features is typically selected to reduce dimensionality and improve model performance. Machine learning-based techniques (e.g., recursive feature elimination or random forest feature importance) are commonly used to identify the most informative features^18^. In total, 1183 features were obtained. To reduce data overfitting and locate the most relevant features, the top 10 features in the training set were identified using minimum redundancy maximum relevance (mRMR). The classifier evaluates feature values and determines the optimal combination of features through repeated iterations. For this purpose, the optimal feature combination was determined based on accuracy using random forest-recursive feature elimination (RF-RFE).

### Radiomics model construction

The patients were randomly divided into a training set (N = 175) and test set (N = 44) (ratio: 8:2). The top 10 features were selected for further examination. Using a standardization method, all selected features were scaled and transformed into the (0, 1) range for preprocessing. After selecting stable and optimal radiomic features using the feature selection, three separate models based on these classifiers were built: KNN, SVM, and LR. The classifier is generally selected according to the data characteristics, sample size, etc. To avoid overfitting the data, three algorithms that performed well in small data sets were used for verification, and their parameter adjustment results are presented in Supplementary Table S2. Grid search was used to determine the optimal hyperparameters based on a given set of values, such as the best k value for KNN was trained in the range of 3–10; the specific parameters are listed in Supplementary Table S2. Five Κ-fold cross-validation was used to improve model performance. Feature extraction based on 2D ultrasound images and clinical information yielded radiomics quality scores, which were calculated from the weighted sum of coefficients of the selected features. The radiomics quality score of our study is presented in Supplementary Table S3. The flowchart of model development is presented in Fig. 1.

### Model evaluation

After building the three models (KNN, SVM, and LR), the diagnostic accuracy of the models was evaluated using the AUC for sensitivity, specificity, MCC, F1 score, and accuracy.

### Statistical analyses

The AUCs were compared using the Delong test. All statistical analyses were performed using SPSS Statistics version 21.0 (IBM Corporation, Armonk, NY, USA). p-values < 0.05 were considered statistically significant. Data normality was evaluated using the Kolmogorov–Smirnov test. Normally distributed continuous variables were compared using Student’s *t*-test and are expressed as means and standard deviations. Categorical variables were compared using the chi-square test and are expressed as frequencies.

## Results

### Demographic and clinical characteristics

Of 219 patients, 114 had T2DM without DN, and 105 had early DN. The mean age of the T2DM and DN groups was 49.75 ± 12.53 and 56.15 ± 10.98 years, respectively (Table 1). We observed no significant differences in age, sex, TC, TG, HDL-C, LDL-C, Apo-A, Apo-B, BUN, CREA, BUN/CREA, UA, or GLU between the two cohorts (Table 2).

**Table 1:** Baseline data for the normal kidney group and the diabetic nephropathy group.

Item	T2DM group (n = 114)	DN group (n = 105)	t/χ2	p
Age, years	49.75 ± 12.53	56.15 ± 10.98	− 4.032	0
Sex, n (%)	65/49	75/30	4.922	0.027
CHO	5.04 ± 1.69	5.13 ± 1.96	− 0.337	0.737
TG	2.74 ± 5.81	2.52 ± 1.92	0.374	0.709
HDL-C	1.11 ± 0.33	1.03 ± 0.38	1.711	0.088
LDL-C	3.08 ± 1.20	3.11 ± 1.53	− 0.163	0.870
Apo-A	1.27 ± 0.25	1.23 ± 0.32	1.157	0.249
Apo-B	1.08 ± 0.33	1.11 ± 0.45	0.479	0.633
APB:APA	0.88 ± 0.33	0.94 ± 0.42	− 0.636	0.526
BUN	5.18 ± 1.46	7.84 ± 3.63	− 7.214	0.000
CREA	64.77 ± 15.91	124.19 ± 65.27	− 9.423	0.000
BUN/CREA	0.08 ± 0.03	0.07 ± 0.02	3.858	0.000
UA	314.09 ± 92.71	402.82 ± 110.57	− 6.453	0.000
GLU	8.99 ± 3.82	8.08 ± 3.54	1.839	0.067

**Table 2:** Baseline data for the training cohorts and the validation cohorts.

Item	Training cohorts (n = 175)	Calidation cohorts (n = 44)	t/χ2	p
Age, years	52.98 ± 12.09	62.70 ± 12.83	0.132	0.895
Sex, n (%)	109/66	31/13	1.017	0.313
CHO	5.12 ± 1.94	4.93 ± 1.24	0.613	0.541
TG	2.70 ± 4.75	2.35 ± 2.52	0.478	0.633
HDL-C	1.05 ± 0.37	1.13 ± 0.29	− 1.338	0.182
LDL-C	3.10 ± 1.42	3.10 ± 1.13	0.026	0.979
Apo-A	1.24 ± 0.29	1.32 ± 0.24	− 1.683	0.094
Apo-B	1.10 ± 0.41	1.07 ± 0.31	0.479	0.633
APB:APA	0.93 ± 0.39	0.84 ± 0.30	1.391	0.166
BUN	6.55 ± 3.16	6.10 ± 2.44	0.872	0.384
CREA	91.46 ± 52.39	100.42 ± 65.43	2.114	0.36
BUN/CREA	0.08 ± 0.03	0.07 ± 0.21	− 0.253	0.801
UA	359.71 ± 109.53	344.39 ± 115.85	0.82	0.413
GLU	8.74 ± 3.83	7.78 ± 3.12	1.549	0.123

### Feature extraction and selection

**Figure 4:**
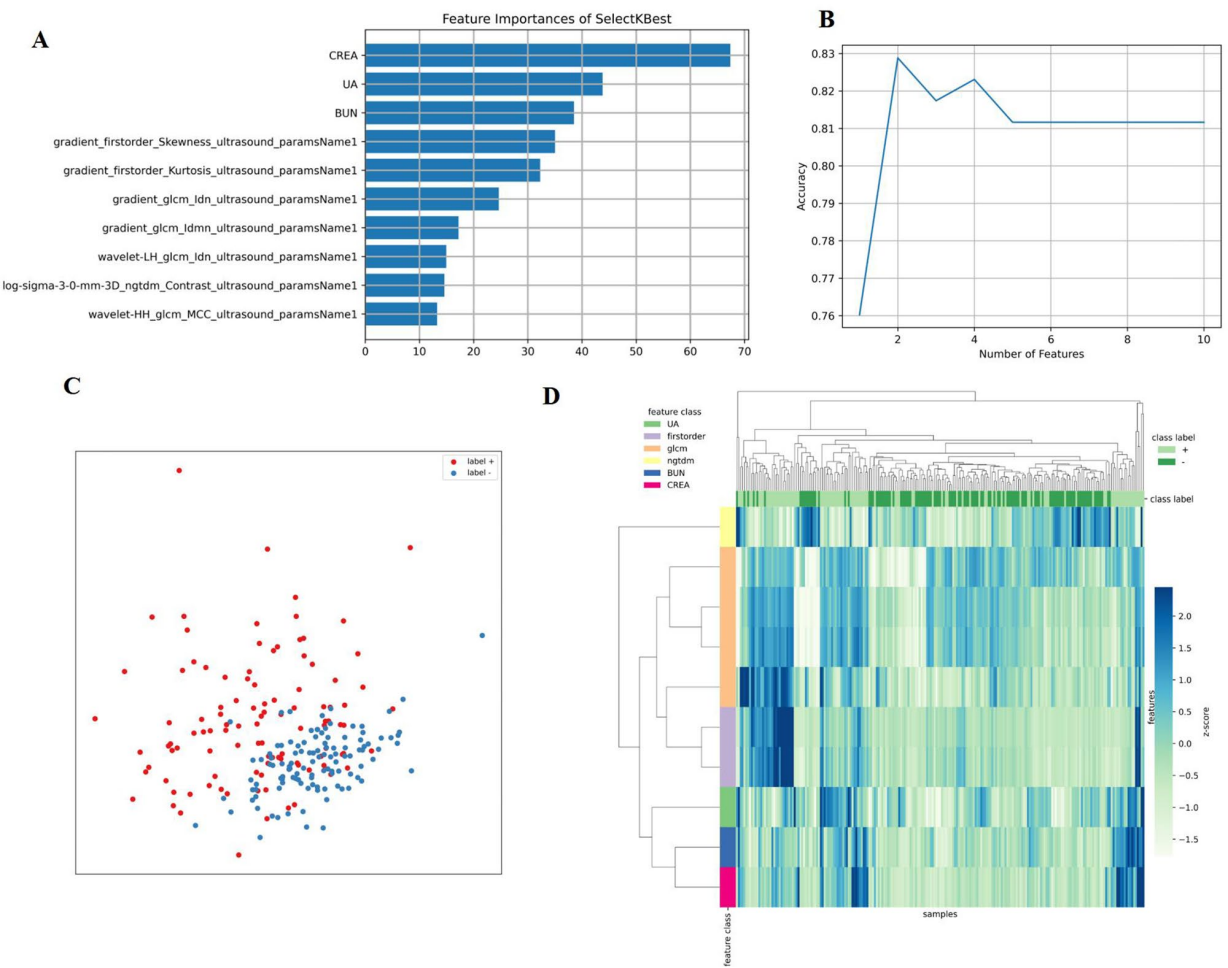
The top 10 properties of the combined model (**A**). The RFE-RF feature selection and distribution of distinct cases on PCA. Step by step, RFE-RF (**B**) was used to determine the optimal feature combination, and the combinations with the highest accuracy would be put into the models. PCA (**C**) demonstrated that the selected features could separate cases in each group intuitively based on their feature values. Heat maps of the features chosen (**D**). The maps' colors represented the value of the specified characteristics.

A total of 1302 radiomics features were extracted from the ultrasound images of each patient. After applying mRMR to the extracted features, the top 10 features were obtained in the training cohort (Fig. 4A). RF-RFE selected the resultant features and determined the best combination of performance features (Fig. 4B). Principal component analysis (PCA) was used to extract the principal components of each group of features and perform dimensionality reduction so that each group of cases could be divided by the eigenvalues. The results are shown in Fig. 4C. The selected features could accurately distinguish between positive and negative cases in the PCA and successfully classify cases with different labels on the left and right sides. The colors in the heat maps correspond to the values of the selected features (Fig. 4D).

### Comparison of the models

**Figure 5:**
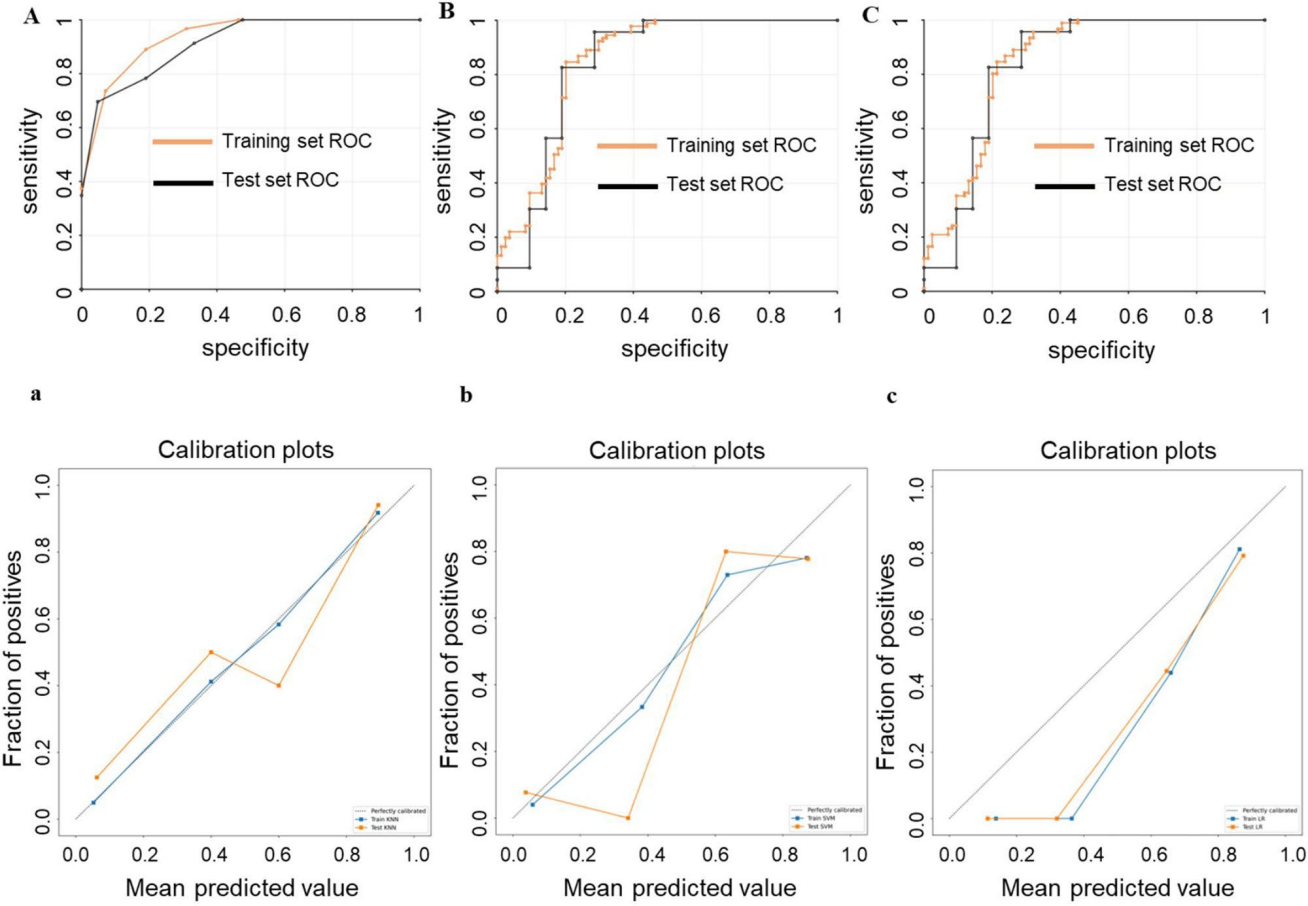
Performance of the radiomics model. Receiver operating characteristic (ROC) curves based on the radiomics models of the KNN (**A**), SVM (**B**), and LR (**C**) classifier, respectively. Calibration curves based on the radiomics model of KNN (**a**), SVM (**b**), LR (**c**) classifier, respectively. The gray diagonal dashed line indicates the perfect predictions, and the solid line indicates the model performance. The solid line is closer to the dashed line, indicating better calibration.

**Figure 6:**
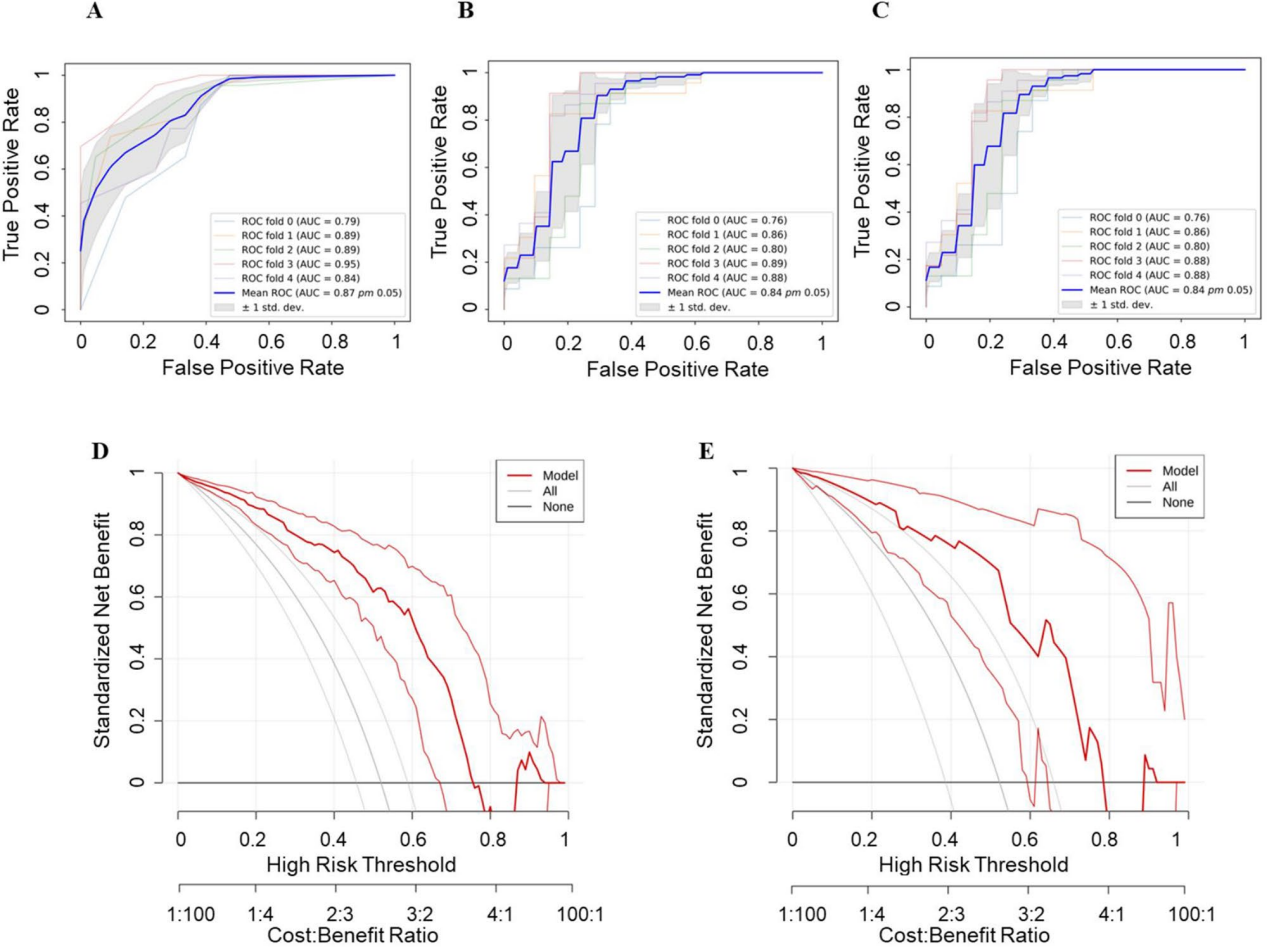
Five-fold cross-validation over the entire dataset results based on different classifiers and decision curve analyses for the combined model. The red line indicates the combined model, the gray line indicates the hypothesis that all patients had diabetic nephropathy, and the black line indicates the hypothesis that no patient had diabetic nephropathy. (**A**) KNN, (**B**) SVM, (**C**) LR, (**D**) Training set, and (**E**) Test set.

The KNN, SVM, and LR radiomics models and respective AUCs are listed in Table 3**.** The AUCs of these models were 0.94, 0.85, and 0.85 in the training cohort and 0.91, 0.84, and 0.84 in the testing cohort (Fig. 5A–C). The calibration curve assesses the agreement between predicted probabilities and observed outcomes from a predictive model: Calibration curves of the models are presented In Fig. 5. The gray dashed line and the solid line indicate complete and estimated prediction, respectively. These lines are close to each other, indicating good performance (Fig. 5a–c). Decision curve analysis decision (DCA) helps evaluate the net benefit of a diagnostic model over a range of decision thresholds. According to the DCA, the use of the radiomics model to predict DN provided a greater net benefit than the “treat all” or “treat none” strategy over a wide range of threshold probabilities, indicating the high clinical applicability of the radiomics model (Fig. 6D, E). Moreover, in the internal five-fold cross-validation, the radiomics model still showed good diagnostic performance (Fig. 6A–C). The results of the Delong test indicated that the KNN model containing ultrasound and clinical features performed better than the other classifiers for early DN diagnosis than the other models. Diagnostic performance in the training and test cohorts is summarized in Table 3.

**Table 3:** Diagnostic performance of different radiomics classifiers in the training cohorts and the validation cohorts of the combined model.

	Classifiers training cohorts							Validation cohorts						
	AUC	SEN	SPE	MCC	F1	ACC	p-value	AUC	SEN	SPE	MCC	F1	ACC	p-value
KNN	0.94	0.89	0.81	0.70	0.86	0.85	0.001	0.91	0.70	0.95	0.66	0.80	0.82	0.159
SVM	0.85	0.85	0.80	0.63	0.83	0.82		0.84	0.96	0.67	0.70	0.86	0.84	
LR	0.85	0.85	0.79	0.66	0.85	0.82		0.84	0.96	0.71	0.70	0.86	0.84	

p-values are from the DeLong’s test; we compared the models under the different classifiers.

In addition, we attempted to build diagnostic model using ultrasound and clinical biochemical data separately. The results of its feature extraction and selection, AUCs, ROC curve, calibration curves of the models, and the DCA are presented in Supplementary Figs. S1–6 and Tables S4, 5.

## Discussion

In this study, we created a radiometric model based on 2D ultrasonography and clinical features to detect early DN. The diagnostic abilities of the KNN, SVM, and LR models containing ultrasound and clinical features were ideal with AUC values all greater than 0.8 in the training and test sets. In particular, the AUC value of this combined model diagnosing early DN based on KNN radiomics reached 0.94, and the DeLong’s test showed the statistical significance of its diagnostic ability. Further, we used clinical data and ultrasound images alone to build diagnostic models. Interestingly, most of their AUC values also exceeded 0.7, especially for the clinical data, showing a great diagnostic advantage. However, comprehensive analysis of AUCs, sensitivity, specificity, MCC, F1 score, accuracy, and DCA revealed that the diagnostic ability of the combined model based on radiomics ultrasound and clinical data was better than that of the clinical data alone or ultrasound images. Thus, we conclude that clinical indicators can complement and reinforce the diagnostic significance of ultrasound indicators.

Long-term hyperglycemia and osmotic diuresis in patients with diabetes can cause glomerular enlargement and hyperfiltration, damaging glomerular capillary endothelial cells, increasing the mesangial matrix, and leading to glomerulosclerosis. Furthermore, hyperglycemia and osmotic diuresis can cause the production and accumulation of extracellular matrix and induce renal fibrosis^26–28^. Although proteinuria and low eGFR can help detect DN, their use as a clinical early diagnostic factor is debatable^29,30^. Ultrasonography is a noninvasive, simple, and low-cost method used clinically to evaluate renal morphology and echogenicity in patients with T2DM^31^. However, many patients with T2DM undergo diagnosis, treatment, and prognostic screening in primary care hospitals, posing a challenge to sonographers’ skills and the recognition of ultrasound imaging. In this context, radiomics extracts imaging features to support diagnostic decision making is significant^12^.

Ke et al.^32^ reported that two color Doppler ultrasound markers, lower intrarenal arterial end-diastolic blood flow velocity and higher arterial resistance index, could accurately diagnose early DN. Bandara et al.^14^ conducted an analysis of the radiomics characteristics of chronic kidney disease based on ultrasound. Their results indicate that Doppler ultrasound has an effective, quantitative index for the diagnosis of early DN, and radiomics is rich and sensitive to extract information on ultrasound images. The application of machine learning and radiomics permitted us to construct excellent diagnostic models from 2D ultrasound images and clinically accessible biochemical indicators. For patients with T2DM, 2D ultrasound and biochemical examinations are routine management schemes, which makes our radiomics model more suitable for the popularization of medical practice. Lee et al.^15^ conducted an analysis of machine learning to assist diagnosis of chronic kidney disease based on ultrasound imaging and found that ultrasound images alone could achieve an AUC of 0.81. Our findings are consistent with their observations, although there were some differences in population selection and disease stage. According to previous reports, the rate of DN misdiagnosis based on clinical information alone is 49.2%^33^. Zou et al.^34^ used machine learning algorithms to build a risk prediction model of ESRD in patients with TD2M and DN and demonstrated that cystatin C, serum albumin, hemoglobin, 24-h urinary protein excretion, and estimated glomerular filtration rate (eGFR) could accurately predict the risk of ESRD. Those results indicate that machine learning based solely on effective clinical data can also diagnose diseases to a large extent. Qu et al.^35^ reported that serum CREA levels had the greatest impact on predicting ESRD in machine learning models. Similarly, in our radiomics model construction, CREA was the largest contribution among all features. However, as in the ultrasound-based multimodal radiomics model developed by Ge et al., for the fibrosis detection of chronic kidney disease, the radiomics model combined with clinical features can offer better diagnostic value^16^.

Classifiers play an important role in machine learning. In our study, three commonly used classifiers were applied to build diagnostic models, and the results revealed that KNN showed the best classification performance. KNN is easier to understand than in all the algorithms, but the category distribution in the sample is very sensitive. The average AUC value of the model was 0.87 after five-fold cross-validation. Compared to the training set with an AUC value of 0.94, despite a slight trend towards an unbalanced data distribution, it still showed excellent diagnostic performance.

Radiomics uses statistical algorithms to quantify medical images. The machine learning section was used for the outcome prediction in the subsequent steps. The combined model we built was better than ultrasound alone or clinical biochemical index model. This may be because the features based on the ultrasound images have a strong specificity for the corresponding disease, such as the texture features that can reflect the disease. Combined with the clinical data, the possible interference of some common diseases is greatly excluded, which creates a good diagnostic performance of our model. In terms of clinical utility, the DCA showed that the radiomics model provided a higher overall net gain.

This study has some limitations. First, the study entailed a single-center design and a small sample, which may not be representative of the entire population. Second, given the retrospective design, we analyzed only 2D ultrasound images and limited clinical data, which inevitably led to selection bias. Nonetheless, we intend to perform a prospective study involving ultrasound elastography imaging and omics data in the future. Third, not all clinical characteristics of patients were included in the analysis. Therefore, larger studies with more features are necessary to construct multiparameter machine learning models and improve the diagnosis of DN.

In conclusion, we established a noninvasive method for diagnosing early DN and demonstrated that the models containing ultrasound imaging and biochemical markers helped diagnose early DN more accurately than the other models. A simple diagnostic tool is beneficial for establishing personalized clinical intervention programs to delay disease progression. Nonetheless, the present findings are only an alternative to clinicians and cannot replace the gold standard of diagnostic biopsy for renal injury.

### Supplementary Information

Supplementary Information.

## Data Availability

The datasets generated or analyzed during the study are available from the corresponding author on reasonable request.
