# Supplementary material for: Value of radiomics-based two-dimensional ultrasound for diagnosing early diabetic nephropathy
Source: Sci Rep. 2023 Nov 22;13:20427. doi: 10.1038/s41598-023-47449-2 (PMC10665410; doi:10.1038/s41598-023-47449-2)
Supplement: Supplementary file 1 — Supplementary Information. [file 41598_2023_47449_MOESM1_ESM.zip › Supplementary Figure1-6+SupplementaryTable1-5/Supplementary Figure1-6.pdf]

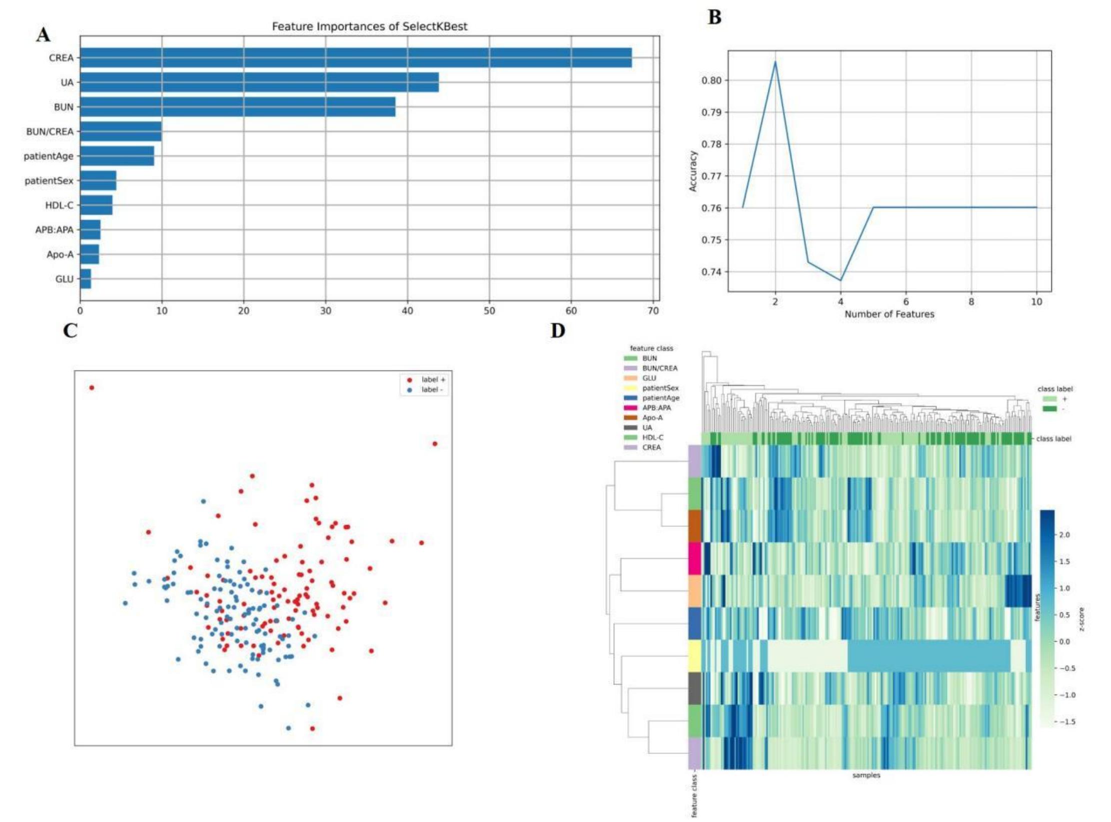

Supplementary Figure 1 The top ten properties of the clinical biochemical model (A). The RFE-RF feature selection and distribution of distinct cases on PCA. Step by step, RFE-RF (B) was used to determine the optimal feature combination, and the combinations with the highest accuracy would be put into the models. PCA (C) demonstrated that the selected features could separate cases in each group intuitively based on their feature values. Heat maps of the features chosen (D). The maps' colors represented the value of the specified characteristics.

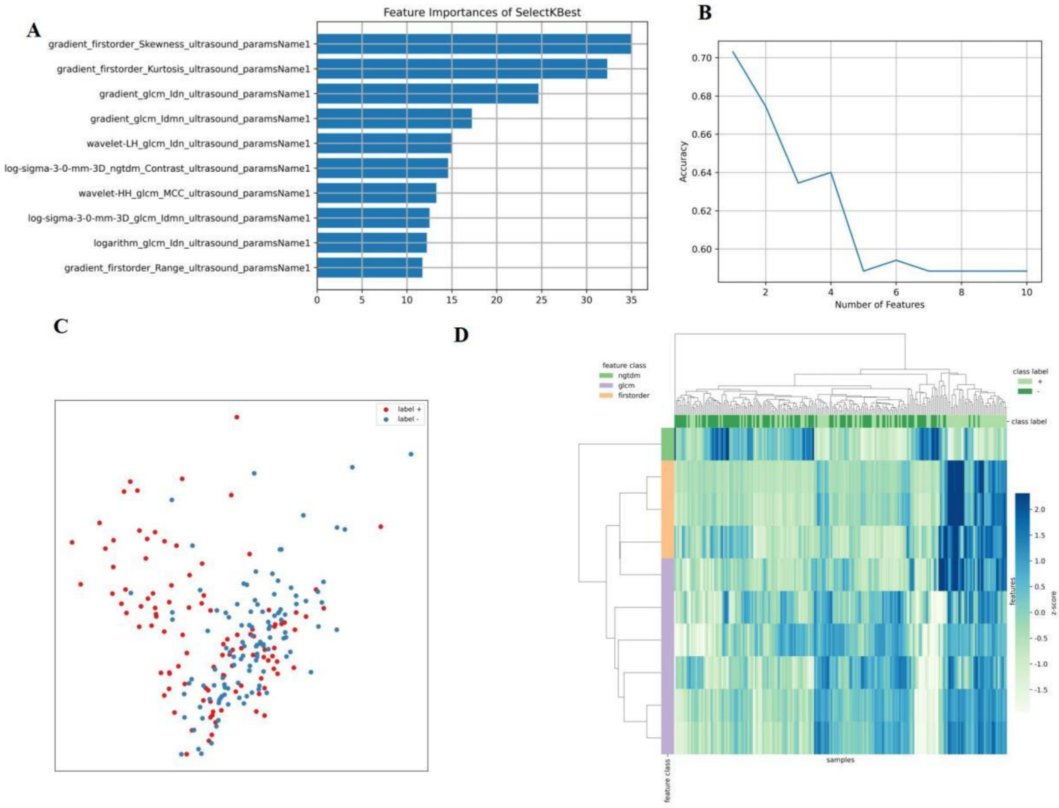

Supplementary Figure 2 The top ten properties of the ultrasound model (A). The RFE-RF feature selection and distribution of distinct cases on PCA. Step by step, RFE-RF (B) was used to determine the optimal feature combination, and the combinations with the highest accuracy would be put into the models. PCA (C) demonstrated that the selected features could separate cases in each group intuitively based on their feature values. Heat maps of the features chosen (D). The maps' colors represented the value of the specified characteristics.

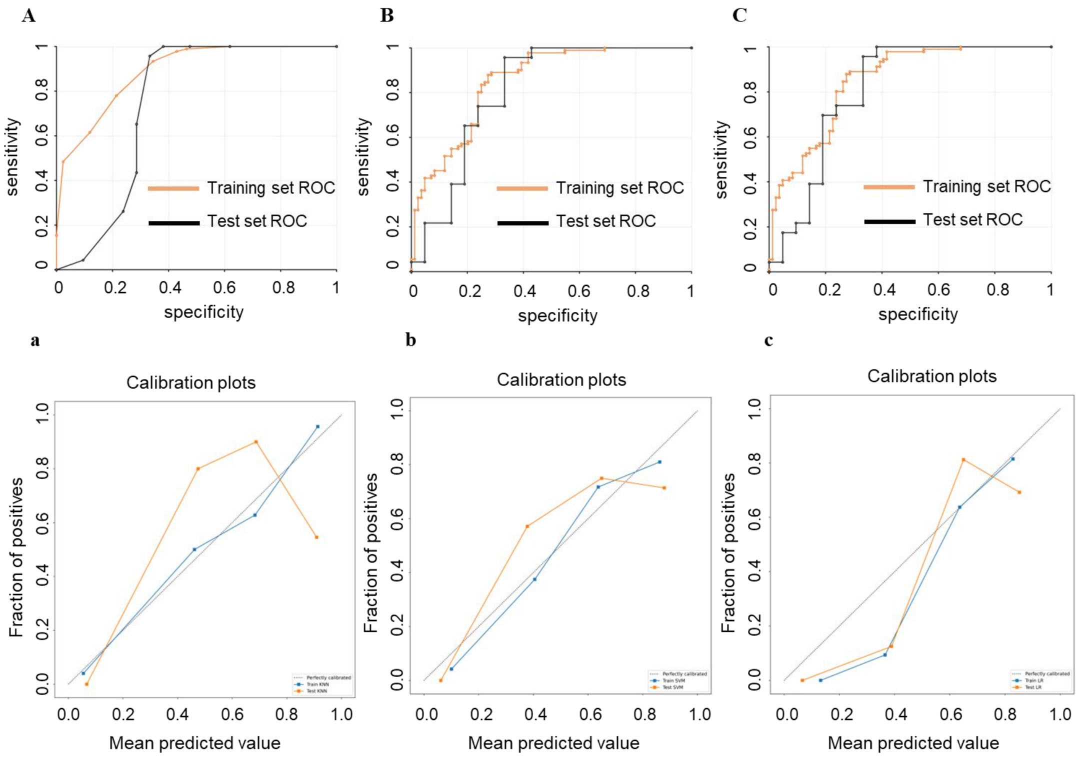

Supplementary Figure 3 Performance of the clinical biochemical model.Receiver operating characteristic (ROC) curves based on the clinical biochemical models of the KNN (A), SVM (B), and LR (C) classifier, respectively. Calibration curves based on the clinical biochemical models of KNN (a), SVM(b), LR(c) classifier, respectively. The gray diagonal dashed line indicates the perfect predictions, and the solid line indicates the model performance. The solid line is closer to the dashed line, which indicates a better calibration.

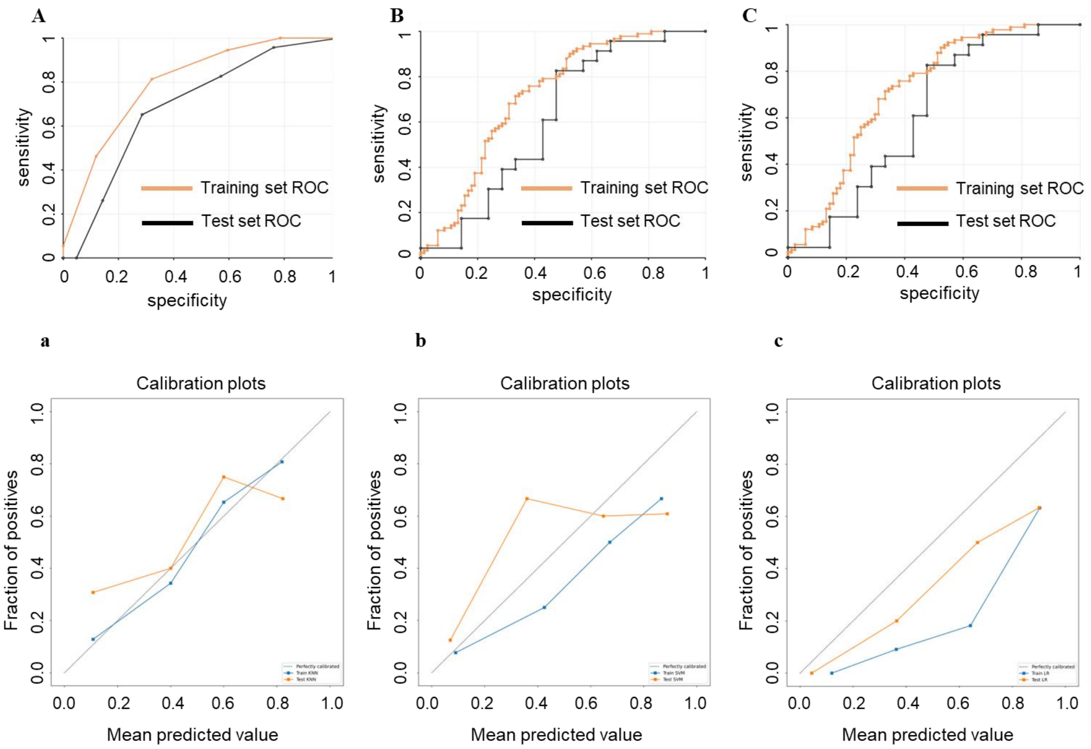

Supplementary Figure 4 Performance of the ultrasound model.Receiver operating characteristic (ROC) curves based on the ultrasound models of the KNN (A), SVM (B), and LR (C) classifier, respectively. Calibration curves based on the ultrasound models of KNN (a), SVM(b), LR(c) classifier, respectively. The gray diagonal dashed line indicates the perfect predictions, and the solid line indicates the model performance. The solid line is closer to the dashed line, which indicates a better calibration.

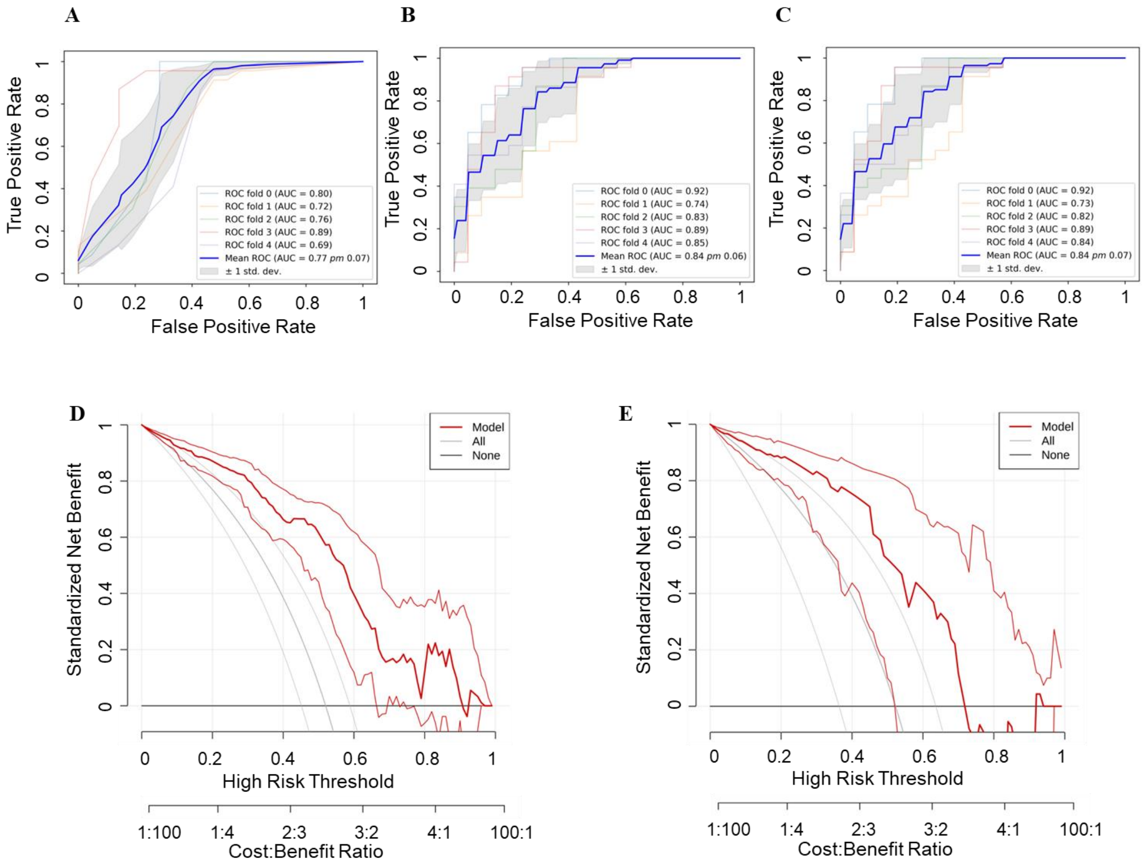

Supplementary Figure 5 5-fold cross-validation over the entire dataset results based on different classifiers and decision curve analyses for the clinical biochemical model.The red line indicates the combined model, the grey line indicates the hypothesis that all patients had diabetic nephropathy, and the black line indicates the hypothesis that no patient had diabetic nephropathy. (A) KNN, (B)SVM, (C)LR, (D) Training set, (E) Test set.

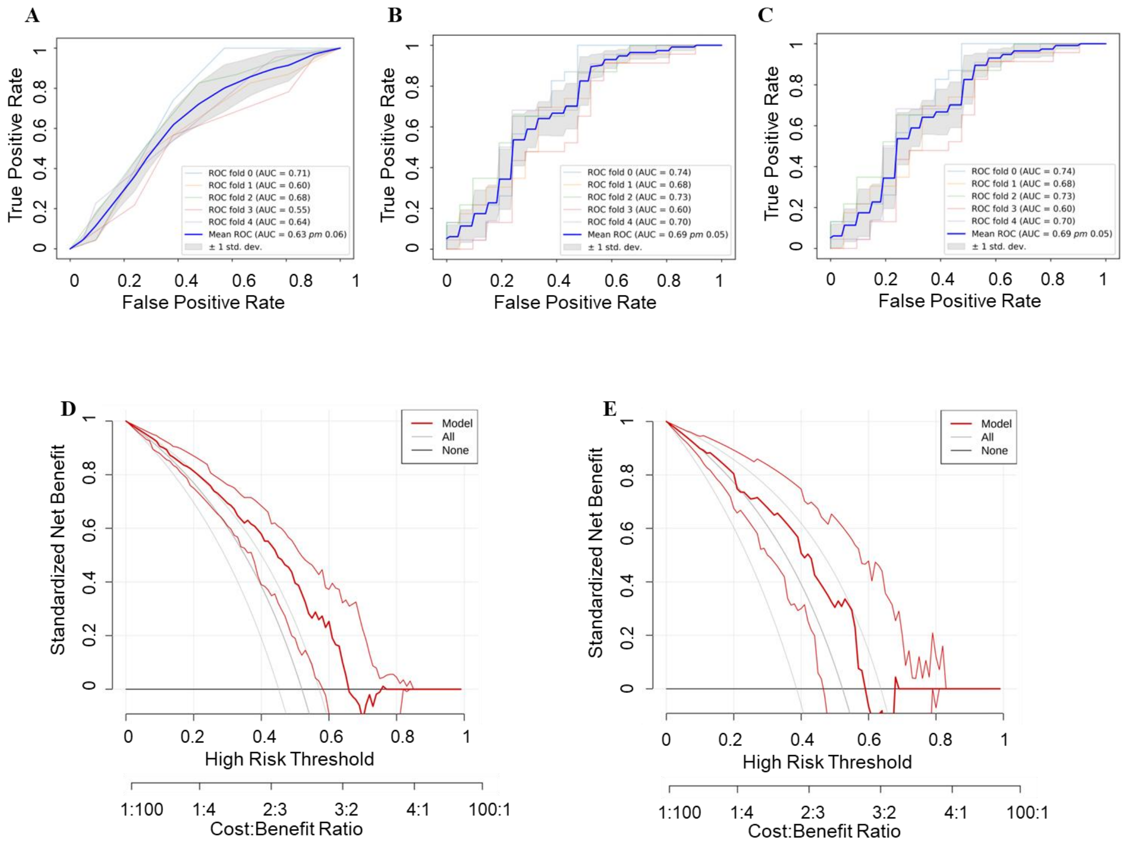

Supplementary Figure 6 5-fold cross-validation over the entire dataset results based on different classifiers and decision curve analyses for the ultrasound model.The red line indicates the combined model, the grey line indicates the hypothesis that all patients had diabetic nephropathy, and the black line indicates the hypothesis that no patient had diabetic nephropathy. (A) KNN, (B)SVM, (C)LR, (D) Training set, (E) Test set.
