# Supplementary material for: Value of radiomics-based two-dimensional ultrasound for diagnosing early diabetic nephropathy
Source: Sci Rep. 2023 Nov 22;13:20427. doi: 10.1038/s41598-023-47449-2 (PMC10665410; doi:10.1038/s41598-023-47449-2)
Supplement: Supplementary file 1 — Supplementary Information. [file 41598_2023_47449_MOESM1_ESM.zip › Supplementary Figure1-6+SupplementaryTable1-5/Supplementary Table S1-5.pdf]

Supplementary Table1. Demographic and clinical biochemical data of all included patients

Value of Radiomics-Based Two-Dimensional Ultrasound for Diagnosing Early Diabetic Nephropathy

Xuee Su, Shu Lin, Yinqiong Huang

patientId	Age	Gender	CHO	TG	HDL-C	LDL-C	Apo-A	Apo-B	APB:APA	BUN	CREA	BUN/CREA	UA	GLU
US201116564	63	male	5.24	1.15	1.04	4.03	1.14	1.3	1.14	7.47	58	0.13	296	5.4
US210811003	52	female	4.62	1.37	0.74	3.51	0.83	1.35	1.63	6.1	69	0.09	362	15.67
US220726122	57	female	5.2	1.36	1.01	3.54	1.24	1.01	0.81	5.12	95.2	0.05	333	4.88
US201203250	63	male	4.17	1	1.09	2.98	1.08	0.94	0.87	5.65	72	0.08	236	4.77
US200411558	57	female	3.72	1.47	1.15	2.15	1.23	0.77	0.63	5.09	53	0.1	332	5.31
US220717389	33	female	3.53	1.4	0.88	2.12	1.13	0.72	0.64	4.14	38.2	0.11	290	4.47
US221018281	31	male	4.88	2.8	0.55	2.53	0.94	1.23	1.31	4.48	164.7	0.03	366	5.09
US230211843	50	male	4.54	1.36	0.89	3.04	1.02	1.08	1.06	8.22	83	0.1	408	7.08
US220907646	33	male	5.55	1.88	1.39	3.4	1.43	1.13	0.79	4.83	84	0.06	294	6.17
US220526437	58	male	3.47	1.29	1.52	1.54	1.4	0.57	0.41	10.45	217	0.05	533	6.65
US220803983	62	male	4.14	4.05	0.61	1.72	0.95	0.87	0.92	9.2	155	0.06	509	8.51
US210505177	56	male	5.01	0.54	1.2	3.56	1.3	1.21	0.93	6.67	69	0.1	227	12.1
US180209699	76	male	4.77	3.55	0.65	2.51	0.54	1.07	1.98	11.61	121.6	0.1	439	11.8
US210810560	60	male	2.85	1.32	0.83	1.41	1.19	0.63	0.53	8.22	114.1	0.07	473	4.82
US210418316	67	male	3.5	1.71	0.66	2.33	0.92	0.85	0.92	3.41	48	0.07	270	4.92
US201120195	51	male	4.88	0.91	1.24	3.26	1.23	1.07	0.87	5.99	63	0.1	233	6.72
US210710982	68	male	3.82	0.7	1.86	1.71	1.86	0.59	0.32	6.49	109	0.06	274	6.55
US190700464	63	female	3.75	2.59	0.28	2.29	0.65	1.24	1.91	3.52	69	0.05	172	11.94
US211020094	73	male	3.92	1.4	1.13	2.41	1.33	0.8	0.6	12.6	197	0.06	338	4.85
US190607869	27	female	3.54	2.16	0.73	1.83	1.06	0.77	0.73	3.31	58.7	0.06	522	12.58
US220214787	60	female	5.15	1.91	1.12	3.38	1.49	1.24	0.83	5.59	57.4	0.1	288	7.53

US190407717	38	female	3.35	1.19	1.18	1.63	1.2	0.68	0.57	3.92	53	0.07	322	10.98
US220922666	64	male	4.89	1.78	0.9	3.09	1.09	1.02	0.94	9.24	159	0.06	256	8.17
US200918601	55	male	4.69	1.29	1.34	2.85	1.37	1.06	0.77	7.3	132	0.06	410	8.42
US210322729	39	male	3.16	1.34	0.9	1.75	1.32	0.63	0.48	7.05	144	0.05	269	5.28
US220215741	52	female	3.46	1.39	0.76	1.84	0.97	0.83	0.86	5.27	91	0.06	206	5.64
US180104332	60	female	5.63	1.83	1.4	3.4	1.67	1.19	0.71	5.13	49	0.1	414	5.26
US180312031	59	female	3.57	0.54	1.09	2.23	1.06	0.76	0.72	3.49	64	0.05	295	3.78
US210112508	61	male	3.61	0.71	1.09	2.45	1.21	0.86	0.71	14.92	196	0.08	501	8.38
US201205001	54	male	5.01	1.82	1	3.43	1.07	1.15	1.07	10.24	135	0.08	562	8.09
US181211212	49	male	9.61	2.14	1.17	7.47	1.27	2.27	1.79	5.3	153	0.03	387	8.05
US210623389	55	male	4.46	1.01	0.83	3.22	1.05	1.16	1.1	2.65	44	0.06	307	10.26
US190907334	56	male	3.31	3.12	0.69	1.57	1.03	0.66	0.64	5.7	108	0.05	376	4.94
US190811709	32	female	4.42	1.51	0.93	2.8	1.17	0.99	0.85	5.1	40	0.13	269	7.53
US221210747	73	female	3.71	1.61	1.05	1.76	1.29	0.77	0.6	7.39	82.6	0.09	270	3.38
US220116113	55	female	4.94	0.99	1.58	3.05	1.63	1.01	0.62	5.17	60.1	0.09	267	9.65
US180204756	46	male	7.97	1.6	1.19	6.05	1.41	1.97	1.4	8.3	103	0.08	329	10.12
US220213611	22	female	7.28	3.23	1.15	5.23	1.33	1.87	1.41	4.41	49	0.09	552	10.91
US210816550	61	male	4.6	1.12	1.2	2.92	1.58	1.03	0.65	6.32	85.3	0.07	360	6.42
US220615592	56	female	5.72	1.58	1.54	3.63	1.55	1.09	0.7	3.89	47	0.08	213	6.3
US220515882	76	female	6.02	0.97	1.75	4.13	1.55	1.4	0.9	6.11	56	0.11	255	7.09
US220301707	53	male	7.71	2.18	1.04	5.92	1.29	2.05	1.59	5.9	90	0.07	400	19.49
US220305173	64	male	3.4	1.06	1.29	1.9	1.44	0.72	0.5	4.09	55	0.07	250	12.62
US220913455	55	male	5.23	2.75	0.71	3.33	0.9	1.19	1.32	7.68	74	0.1	416	10.35
US220104111	50	male	5.48	1.73	1.14	3.5	1.37	1.31	0.96	9.96	148.7	0.07	434	7.53
US210604713	53	male	8.34	0.95	3.4	4.87	3.02	1.46	0.48	7.34	81	0.09	306	6.35
US210514599	56	female	8.8	1.47	1.56	6.66	1.76	1.91	1.09	4.19	49	0.09	211	8.66

US210914124	26	male	9.98	32.79	0.5	1.23	0.79	1.02	1.29	3.7	76	0.05	373	18.75
US200502859	72	male	2.85	1.25	0.96	1.62	0.98	0.57	0.58	5.22	108	0.05	306	5.59
US211101227	58	female	4.11	2.16	0.96	2.39	1.35	0.92	0.68	5.75	49	0.12	255	7.15
US180201385	60	female	8.14	1.58	2.14	5.28	1.88	1.55	0.82	6.09	49	0.12	346	8.15
US180517636	62	female	3.2	1.23	1.09	1.55	0.98	0.77	0.79	2.5	70	0.04	247	6.88
US190611443	56	female	4.6	1.21	1.11	2.94	1.26	1.11	0.88	4.4	64	0.07	330	3.93
US200701048	58	male	2.52	2.31	0.65	1.14	1	0.56	0.56	4.23	98	0.04	494	5.63
US211109007	71	male	4.87	2.92	0.74	3.31	1	1.24	1.24	4.54	98	0.05	338	4.41
US211126786	57	female	6.49	1.65	1.47	4.64	1.58	1.42	0.9	3.7	52.4	0.07	321	10.23
US191215162	36	male	4.84	4.5	0.84	2.85	1.1	1.12	1.02	6	84	0.07	392	9.86
US221006528	56	male	6.37	4.67	1.02	3.2	1.35	0.81	0.6	3.99	93	0.04	359	7.88
US211207622	61	female	4.88	0.94	1.5	3.02	1.59	0.84	0.53	7.34	60.4	0.12	311	4.84
US210701158	56	male	3.96	2.16	0.88	2.52	1.23	0.94	0.76	6.46	73	0.09	331	8.81
US230413488	56	male	3.32	2.91	0.54	1.26	1	0.7	0.7	18.85	241.1	0.08	415	5.98
US220813078	51	male	5.17	3.17	0.97	3.17	1.28	1.14	0.89	12.74	166	0.08	680	5.08
US220111332	62	male	6.13	2.12	0.8	4.59	1	1.58	1.58	5.9	71	0.08	278	12.41
US210421312	54	male	6.17	0.98	1.16	4.6	1.24	1.44	1.16	6.9	75	0.09	370	8.46
US221002887	62	male	6.63	4.01	0.92	4.1	1.32	1.49	1.13	6.69	85	0.08	533	12.71
US211218890	53	female	6.38	0.95	1.72	4.37	1.73	1.21	0.7	7.39	62	0.12	362	8.86
US190721871	61	female	5.06	2.22	0.77	3.28	1.06	1.32	1.25	3.18	42	0.08	379	16.95
US211008285	40	male	2.87	1.43	1.17	1.47	1.26	0.6	0.48	7.9	73	0.11	352	5.51
US220717192	56	male	2.34	2.44	0.38	1.12	0.68	0.64	0.94	14.43	152	0.09	557	5.76
US211207992	44	male	5.59	1.41	0.89	4.47	0.98	1.44	1.47	5.31	87	0.06	320	11.64
US220203738	63	male	5.51	1.35	1.45	3.5	1.34	1.3	0.97	10.3	98	0.11	389	16.74
US210505475	26	female	5.09	2.22	1.03	3.4	1.26	1.16	0.92	3.51	41.6	0.08	419	9.26
US211107782	57	male	3.62	1.9	1.26	1.84	1.48	0.63	0.43	3.38	79.8	0.04	297	10.83

US211015111	54	male	5.26	6.46	0.69	1.86	1.19	1.01	0.85	9.88	87	0.11	396	6.09
US210314340	50	female	6.17	7.46	0.76	2.88	1.25	1.38	1.1	5.78	98	0.06	481	7.3
US220217375	35	male	5.29	1.54	1	3.58	1.15	1.22	1.06	4.14	70.1	0.06	371	6.35
US210604755	45	male	4.06	1.12	1.04	2.69	1.31	0.93	0.71	5.75	88	0.07	435	6.21
US190411927	60	female	4.42	7.39	0.58	0.48	1.12	0.74	0.66	4.1	58.1	0.07	430	15.41
US190823784	44	male	7.12	9.78	0.89	2.72	1.49	1.27	0.85	3.89	58	0.07	367	8.92
US220108782	50	male	6.35	3.92	0.93	4.23	1.19	1.63	1.37	8.5	85	0.1	432	10.56
US220722549	55	female	3.29	3.43	0.76	1.46	1.05	0.73	0.7	5.63	43	0.13	359	7.54
US220122972	34	male	4.5	0.93	0.93	3.08	1.03	0.97	0.94	5.08	62.7	0.08	250	12.95
US220919675	47	male	4	1.13	0.95	2.48	1.01	0.86	0.85	6.64	81	0.08	267	6.22
US200722043	66	female	2.55	0.58	1.19	1.07	1.28	0.46	0.36	8.65	40	0.22	350	7.19
US200906296	53	male	4.18	7.99	0.67	1.83	1.01	0.92	0.91	6.97	69	0.1	335	13.58
US220717946	51	male	4.87	1.66	1.83	2.61	1.64	0.78	0.48	4.84	65.6	0.07	391	8.5
US220722644	31	female	3.72	1.83	1.33	1.91	1.63	0.75	0.46	3.07	44	0.07	257	6.55
US211210466	58	male	5.19	1.43	1.08	3.52	1.27	1.12	0.88	6.6	72.9	0.09	387	5.18
US210513374	56	female	12.8	36.18	0.41	1.5	0.9	1.29	1.43	3.8	52.3	0.07	240	17.49
US191211700	58	male	2.97	2.71	0.58	1.32	0.86	0.7	0.81	8.99	173	0.05	369	8.78
US210215646	53	male	4.8	0.92	1.3	3.45	1.32	1.03	0.78	5.26	72	0.07	388	4.97
US220803751	39	male	2.71	1.26	0.75	1.63	0.84	0.63	0.75	6	104	0.06	402	6.6
US230222536	39	male	6.35	5.76	1.24	3.85	1.83	1.44	0.79	4.7	96	0.05	512	19.16
US210409663	28	male	5.7	4.01	0.75	3.52	1.15	1.31	1.14	10.2	196	0.05	496	6.52
US221016202	73	female	2.7	1.03	0.81	1.42	0.75	0.56	0.75	9.46	162.3	0.06	341	5.31
US220107653	79	female	4.52	0.78	1.08	3.08	1.11	0.86	0.77	7.45	113.1	0.07	450	6.47
US220311788	62	female	4.36	1.94	0.93	2.72	1.23	1.04	0.85	5.6	74	0.08	270	19.57
US220814388	38	male	3.58	0.85	0.98	2.4	1.04	0.78	0.75	5.4	73	0.07	453	6.64
US220302786	72	female	10.16	3.62	1.3	7.08	1.49	2.46	1.65	11.1	299	0.04	343	5.97

US201213010	64	female	3.64	1.52	1.24	2.21	1.32	0.79	0.6	6.7	39	0.17	393	8.06
US211120478	32	female	6.06	1.83	1.6	4.12	1.67	1.28	0.77	5.66	56.7	0.1	228	7.46
US220211155	58	female	5.96	0.82	1.21	4.25	1.33	1.5	1.13	3.55	60	0.06	237	15.72
US221008916	70	female	3.32	1.56	1.32	1.54	1.36	0.58	0.43	4.4	73	0.06	318	6.81
US221008922	73	male	4.02	0.65	1.23	2.61	1.31	0.86	0.66	6.5	75	0.09	384	7.59
US200418577	42	male	3.94	0.98	1	2.55	1.17	0.92	0.79	7.31	94	0.08	259	5.69
US210603509	32	female	5.54	2.85	0.79	3.89	1.05	1.36	1.3	5.42	44	0.12	318	6.12
US200713321	32	male	4.64	1.51	0.63	3.53	0.72	1.38	1.92	4.04	65	0.06	596	10.82
US200411824	43	female	7.15	3.35	1.23	5.09	1.45	1.65	1.14	2.86	41	0.07	307	15.06
US220500797	62	female	8.16	1.79	0.93	6.61	0.94	2.28	2.43	12.65	123	0.1	499	4.24
US200904693	61	male	5.19	0.54	1.12	3.63	1.13	1.29	1.14	5.93	71	0.08	230	11.78
US221124169	56	male	13.05	40.1	0.47	0.33	0.88	0.74	0.84	3.54	73.7	0.05	323	7.57
US220218111	62	female	3.93	0.94	0.89	2.38	0.88	0.94	1.07	5.51	47.5	0.12	76	7.99
US190801987	59	female	2.89	2.64	0.8	0.89	1.12	0.61	0.54	3.11	47	0.07	457	8.25
US220619400	48	male	4.64	0.59	1.22	3.06	1.46	0.99	0.68	7.72	79.1	0.1	350	4.75
US220605797	54	male	4.09	1.32	0.98	2.63	1.16	0.97	0.84	6.47	167	0.04	415	5.56
US211020113	44	female	4.56	1.71	1.44	2.69	1.49	0.8	0.54	6.98	30.8	0.23	184	10.72
US200815109	57	male	5.65	1.68	1.26	4	1.5	1.38	0.92	3.36	65	0.05	326	10.63
US190408569	57	female	5.14	1.64	0.83	3.56	0.83	1.4	1.69	4.6	68	0.07	231	4.28
US220708196	63	male	4.72	1.23	1.5	2.85	1.53	0.88	0.58	5.37	72	0.07	280	7.2
US210422233	39	male	4.62	2.81	0.71	2.91	1.01	1.05	1.04	4.6	87	0.05	347	6.35
US220819570	58	male	4.08	2.29	0.66	2.35	0.92	0.95	1.03	7.06	104	0.07	546	4.6
US221015128	43	male	4.16	0.87	0.86	2.72	0.96	0.91	0.95	4.51	61	0.07	458	6.7
US211117617	54	female	4.19	0.59	1.78	2.38	1.66	0.77	0.46	3.08	35	0.09	170	7.19
US210715753	45	male	5.77	2.56	0.77	4.17	1.13	1.53	1.35	9.28	123	0.08	390	5.51
US220714488	59	female	6.6	1.37	1.56	4.54	1.59	1.26	0.79	4.85	45.3	0.11	242	5.34

US221107547	62	male	4.31	2.21	0.9	2.85	1	0.96	0.96	11.69	194	0.06	340	21.91
US200921512	49	male	2.67	1.24	1.02	1.33	1.05	0.56	0.53	6.44	67	0.1	317	6.82
US210616105	53	female	7.06	3.16	1.03	5.11	1.46	1.67	1.14	5.23	50.3	0.1	390	9.47
US221005096	53	male	2.66	3.14	0.71	1.25	1.14	0.6	0.53	5.07	57.7	0.09	476	10.99
US220619266	59	male	5.6	3.35	0.77	3.34	1.13	1.33	1.18	11.19	296.1	0.04	717	9.71
US200400314	29	female	4.79	1.25	1.18	3.26	1.27	1	0.79	3.64	60	0.06	405	5.19
US211112649	38	male	6.52	2.39	1.05	4.9	1.33	1.56	1.17	4.56	82.4	0.06	295	12.7
US220116313	58	male	4.5	1.61	0.95	2.49	1.14	0.63	0.55	9.86	134	0.07	461	9.92
US210700463	60	male	4.29	1.73	0.68	2.18	1.07	0.7	0.65	8.98	206.3	0.04	297	11.95
US220606538	40	female	4.61	1.31	0.74	3.14	1.07	1.07	1	3.41	56	0.06	318	10.46
US191122610	53	male	4.91	0.95	1.1	3.83	1.23	1.11	0.9	6.18	98	0.06	463	6.15
US181012678	59	male	4.99	1.56	0.94	3.34	1.2	1.32	1.1	5.08	72	0.07	291	10.7
US220709337	59	male	5.58	1.47	0.84	4.37	0.99	1.5	1.52	6.97	81	0.09	465	12.03
US220303695	31	female	6.18	3.4	1.19	3.73	1.38	1.4	1.01	4.09	52.2	0.08	402	9.88
US220118683	47	female	7.79	1.54	1.51	5.72	1.52	1.82	1.2	6.08	63	0.1	369	15.06
US210704843	46	male	3.43	1.33	0.83	2.15	1.15	0.8	0.7	3.46	53.4	0.06	194	8.26
US230316067	57	male	4.4	2.54	1.92	1.62	2	0.75	0.38	5.9	110.2	0.05	648	12.78
US211005658	54	male	4.33	3.6	0.88	2.16	1.2	0.95	0.79	6.17	88	0.07	392	7.79
US210909966	37	male	5.36	8.48	0.58	1.56	1.08	0.94	0.87	4.44	77.5	0.06	646	9.34
US210608580	31	female	3.03	3.16	0.78	1.39	1.37	0.69	0.5	3.81	40	0.1	290	6.48
US200810633	38	male	4.19	1.79	0.93	2.66	1.23	0.98	0.8	5.31	80	0.07	306	6.39
US210311924	53	male	8.97	2.64	1.24	6.86	1.44	1.99	1.38	6.25	107	0.06	353	7.52
US190613989	55	male	5.42	0.86	1.33	3.7	1.36	0.97	0.71	5.02	90.6	0.06	326	6.49
US181100938	48	female	2.98	1.95	0.99	1.1	1.23	0.33	0.27	5.72	60	0.1	429	7.53
US221112726	68	female	4	1.12	1.05	2.69	0.89	0.93	1.04	8.37	135	0.06	428	10.61
US220502516	41	female	9.1	5	0.88	5.25	1.16	1.7	1.47	15.5	259	0.06	481	10.06

US220724348	68	male	3.72	1.11	0.98	2.05	1.14	0.75	0.66	8.87	112.4	0.08	483	9.89
US220622017	58	male	3.24	0.71	1.41	1.49	1.34	0.5	0.37	5.32	89.4	0.06	252	6.19
US190106738	39	male	9.79	2.25	0.61	8.16	0.86	2.49	2.9	5.91	61	0.1	263	10.25
US221013276	63	male	6.41	1.21	0.73	5.15	0.93	1.74	1.87	12.32	99	0.12	522	5.85
US211114165	66	female	4.74	1.21	1.01	3.56	1.15	1.11	0.97	5.17	59	0.09	340	5.72
US220828247	60	male	3.63	1.2	0.88	2.31	1.01	0.81	0.8	7.75	134	0.06	366	5.95
US220111401	71	male	5.49	0.96	1.84	3.36	1.65	1.11	0.67	5.4	83	0.07	219	10.45
US210916914	66	female	7.19	1.28	1.42	5.42	1.5	1.53	1.02	6.78	73	0.09	256	17.87
US200608931	37	male	3.18	1.14	1.32	1.6	1.26	0.68	0.54	4	57	0.07	275	8.19
US210718525	49	female	5.99	1.45	0.98	4.53	1.24	1.59	1.28	1.81	46	0.04	169	15.41
US210407057	64	female	5.35	1.81	1.24	3.81	1.5	1.28	0.85	6.7	57	0.12	500	6.26
US220600362	26	female	3.52	1.39	0.81	2.12	1.1	0.78	0.71	8.02	36.9	0.22	219	9.66
US220819565	61	female	6.73	0.98	1.48	4.76	1.5	1.35	0.9	8.22	51	0.16	196	3.76
US221114700	67	male	4.69	1.91	0.88	2.76	1.13	1.18	1.04	12.2	212	0.06	349	7.37
US221105049	73	male	3.13	1.84	0.68	1.73	0.92	0.73	0.79	6.05	98.7	0.06	507	3.87
US220505720	58	male	5.99	1.76	1.15	3.9	1.4	1.37	0.98	6.03	71.2	0.08	348	18.67
US220111338	53	male	4.6	3.53	0.67	2.72	0.86	1.02	1.19	5.94	60.1	0.1	302	8.18
US220212153	55	male	12.05	7.62	0.97	6.82	1.4	2.46	1.76	7	148	0.05	409	12.11
US220714837	64	male	5.66	2.67	1.21	3.4	1.42	1.25	0.88	27.5	297	0.09	653	14.26
US211110194	58	male	4.55	0.89	0.84	3.66	0.96	1.12	1.17	6.66	59	0.11	242	9.44
US190809485	57	female	5.32	4.19	1.03	2.39	1.35	0.95	0.7	11.9	161	0.07	532	6.6
US220203577	33	male	12.37	8.01	1.1	5.3	1.64	1.51	0.92	9.3	240	0.04	397	22.1
US211208656	47	male	6.55	1.27	1.06	4.92	1.24	1.49	1.2	5.6	80.6	0.07	155	7.73
US191215437	35	male	3.77	1.31	1.71	1.82	1.73	0.58	0.34	5.58	71	0.08	374	7.35
US220820189	50	male	3.27	1.23	0.89	1.78	1.12	0.71	0.63	5.59	102	0.05	530	5.94
US210115086	71	male	2.22	0.94	1.24	0.7	1.28	0.36	0.28	7.96	61	0.13	350	6.62

US210211413	59	male	5.06	2.04	1.13	3.44	1.46	1.14	0.78	6.5	64	0.1	319	6.61
US220309444	40	male	7.37	14.38	0.7	1.41	1.09	1	0.92	3.83	59.3	0.06	200	9.24
US230417332	73	female	5.79	7.44	0.96	2.07	1.64	1.12	0.68	5.44	62	0.09	475	11.72
US200910966	55	female	7.4	2.47	1.26	5.48	1.42	1.85	1.3	5.37	77	0.07	410	5.45
US201203250	63	male	4.17	1	1.09	2.98	1.08	0.94	0.87	5.65	72	0.08	236	4.77
US220312362	53	male	5.7	0.88	0.95	4.21	0.97	1.38	1.42	5.65	60.8	0.09	236	5.93
US191122154	32	male	4.64	0.68	1.17	3.78	1.17	1.02	0.87	1.92	62	0.03	427	8.53
US221113253	60	male	3.97	1.59	0.92	2.52	1.08	0.87	0.81	5.06	131	0.04	178	5.09
US230207824	53	male	4.48	1.5	1.29	2.72	1.45	0.89	0.61	3.93	61.8	0.06	268	7.84
US220825877	68	female	4.64	2.03	0.98	2.94	1.27	1.05	0.83	9.47	129.4	0.07	286	5.15
US211017918	36	male	7.46	2.86	1.26	5.56	1.43	1.76	1.23	5	71	0.07	330	9.03
US211206484	55	male	4.02	0.66	1.61	2.43	1.5	0.8	0.53	5.86	80	0.07	412	5.89
US221004027	60	male	4.07	2.2	1.53	1.96	1.64	0.69	0.42	6.43	113.8	0.06	522	7.81
US210900232	42	female	4.04	0.82	1.63	2.27	1.59	0.68	0.43	4.61	50	0.09	155	6.83
US220507455	47	male	2.43	1.39	0.88	1.08	1.08	0.53	0.49	3.32	58	0.06	254	5.63
US201209258	58	female	3.79	2.39	0.84	2.46	1.16	1.02	0.88	5.23	66	0.08	194	6.6
US191202214	53	male	4.94	1.8	0.92	4.05	1.09	1.22	1.12	4.23	94	0.05	316	12.59
US211217850	62	male	7.18	1.32	1.94	4.89	1.96	1.45	0.74	9.45	203.9	0.05	311	7.7
US210916248	56	female	5.93	0.98	1.79	4.22	1.79	1.33	0.74	4.8	44	0.11	116	18.77
US190812647	51	male	6.17	2.52	0.94	4.5	1.15	1.39	1.21	5.51	67	0.08	362	8.51
US220804134	58	female	6.46	1.12	1.05	4.82	1.3	1.42	1.09	4.91	63.8	0.08	335	5.34
US220705214	62	male	4.81	1.9	1.29	2.85	1.12	0.93	0.83	10.02	273	0.04	406	5.13
US221214304	53	male	5.76	1.35	1.34	3.8	1.49	1.24	0.83	9.88	228.5	0.04	424	7.73
US230415872	28	male	5.23	1.58	0.9	3.67	1.11	1.11	1	4.6	58.3	0.08	398	7.75
US221014606	72	female	4.05	1.97	1.08	2.2	1.42	0.84	0.59	4.74	52.2	0.09	266	5.5
US191101916	52	female	4.15	1.71	1.1	2.81	1.33	0.92	0.69	4.1	56	0.07	370	8.27

US230211851	55	male	4.98	1.36	0.92	3.76	0.99	1.31	1.32	4.55	63.7	0.07	279	8.87
US200503644	59	male	5.16	2.1	0.91	3.93	1.18	1.39	1.18	6.22	78	0.08	359	9.52
US221011078	54	male	4.05	2.48	1.04	2.18	1.48	0.87	0.59	12.55	175.4	0.07	633	9.4
US201111927	47	female	5.94	1.73	1.05	4	1.11	1.41	1.27	5.1	47	0.11	359	7.4
US220625279	54	male	6.63	9.46	0.97	3.8	1.23	1.29	1.05	6.9	104	0.07	486	9.66
US211122291	29	female	4.75	1.65	1.12	3.12	1.48	1.06	0.72	3.64	47.9	0.08	426	7.62
US200812664	55	male	4.2	1.99	1.4	2.1	1.66	0.84	0.51	9.07	327.7	0.03	265	5.01
US220704009	68	male	5.31	2.07	1.32	2.92	1.6	0.98	0.61	10.3	205	0.05	453	5.16
US220312514	32	male	4.11	5.79	0.67	1.63	1.14	0.77	0.68	4.95	71.3	0.07	300	15.92
US220908934	31	male	5.4	1.74	0.88	4.21	0.98	1.44	1.47	4.53	61	0.07	309	15.88
US210607200	57	female	4.31	0.76	1.55	2.67	1.75	0.84	0.48	10.83	154	0.07	220	6.22
US211110157	61	male	4.43	0.85	1.19	2.83	1.32	0.96	0.73	8.11	142.7	0.06	370	4.85
US210511424	44	male	5.94	4.45	0.81	3.31	1.17	1.4	1.2	5.16	104	0.05	418	8.12
US190822570	79	female	3.39	1.46	0.78	2.11	0.98	0.88	0.9	2.02	49	0.04	153	6.16
US191107280	50	male	5.65	1.14	1.33	3.92	1.34	1.29	0.96	10.22	193	0.05	533	6.69
US211202461	22	male	3.54	1.61	1.22	2.01	1.33	0.64	0.48	5.22	73	0.07	504	3.97

Supplementary Table 2. The parameters used in the construction of the model

Classifier	Parameters	
SVM	kernel	rbf
	degree	3.00
	gamma	scale
	Coef 0	0.00
	C	1.00
KNN	n_neighbors	5.00
Logistic Regression	C	1.00
	penalty	l2

Rbf radical basis function KNN K-nearest neighbor SVM Support vector machine

Supplementary Table 3. Radscore of different radiomics classifiers of the models

Classifiers	Radscore
The combined model	
SVM	-4.591*CREA-2.770*gradient_firstorder_Kurtosis_ultrasound_paramsName1+
LR	-5.749*CREA-3.364*gradient_firstorder_Kurtosis_ultrasound_paramsName1+

The clinical biochemical model	
SVM	-3.952*CREA-3.031*UA+2.302
LR	-3.831*CREA-2.593*UA+1.970
The ultrasound model	
SVM	-4.354*gradient_firstorder_Kurtosis_ultrasound_paramsName1+1.166
LR	-3.522*gradient_firstorder_Kurtosis_ultrasound_paramsName1+0.536

KNN K-nearest neighbor   SVM Support vector machine   LR Logistic Regression

Supplementary Table 4. Diagnostic performance of different radiomics classifiers in the training cohorts and the validation cohorts of the clinical biochemical model

Classifiers	training cohorts							validation cohorts						
	AUC	SEN	SPE	MCC	F1	ACC	p-value	AUC	SEN	SPE	MCC	F1	ACC	p-value
KNN	0.89	0.93	0.66	0.62	0.83	0.80	0.003	0.74	0.95	0.67	0.66	0.85	0.82	0.304
SVM	0.85	0.87	0.73	0.61	0.83	0.81		0.81	0.96	0.67	0.66	0.85	0.82	
LR	0.85	0.87	0.73	0.61	0.83	0.81		0.81	0.96	0.67	0.66	0.85	0.82	

The p-values were from the DeLong's test, and we compared the models under the different classifiers

Supplementary Table 5. Diagnostic performance of different radiomics classifiers in the training cohorts and the validation cohorts of the ultrasound model

Classifiers	training cohorts							validation cohorts						
	AUC	SEN	SPE	MCC	F1	ACC	p-value	AUC	SEN	SPE	MCC	F1	ACC	p-value
KNN	0.80	0.81	0.63	0.50	0.77	0.75	0.002	0.70	0.65	0.71	0.37	0.68	0.68	0.279
SVM	0.72	0.71	0.67	0.38	0.71	0.69		0.62	0.83	0.53	0.37	0.73	0.68	
LR	0.72	0.71	0.67	0.38	0.71	0.69		0.62	0.83	0.48	0.32	0.72	0.66	

The p-values were from the DeLong's test, and we compared the models under the different classifiers
